# Multiple Simultaneous Rare Distant Metastases as the Initial Presentation of Papillary Thyroid Carcinoma: A Case Report

**DOI:** 10.3389/fendo.2019.00759

**Published:** 2019-11-08

**Authors:** Jing Yang, Yu Ma, Yanping Gong, Rixiang Gong, Zhihui Li, Jingqiang Zhu

**Affiliations:** Thyroid and Parathyroid Surgery Center, West China Hospital, Sichuan University, Chengdu, China

**Keywords:** PTC, rare distant metastases, 18F-FDG-PET/CT, palliative therapy, chemotherapy

## Abstract

Papillary thyroid carcinoma (PTC) commonly metastasizes to regional lymph nodes. However, they infrequently cause rare distant metastases (RDMs), with the exclusion of lungs and bone metastases. RDMs are seldom identified prior to a primary thyroid cancer diagnosis. Therefore, cases initially presenting with synchronously multiple RDMs from PTC are extremely infrequent. This is a rare case of a 48-year-old man with initial diaphragm, pancreatic, and liver tumors from PTC. Following resection of the tumors, an ultrasound-guided fine-needle aspiration (US-FNA) cytology of a mass in the thyroid's left lobe revealed PTC. After postoperative recovery for more than 4 months, physical examination identified an irregular large nodule in the thyroid's isthmus and left lobe, a swollen lymph node in the left neck, and a mass in the right parotid gland. Ultrasound reexamination revealed numerous hypoechoic masses as follows: one in the thyroid's isthmus and entire left lobe (7.3 × 5.9 × 5.1 cm) and multiple in the thyroid's right lobe (0.2–0.3 cm). Ultrasound examination also showed several swollen lymph nodes in the left neck, a mass in the left gluteus maximus, and several masses in both the bilateral parotid and salivary region. The US-FNA's pathological examination confirmed metastatic PTCs in the left gluteus maximus and bilaterally located in the parotid and salivary gland. 18-fluorodeoxyglucose positron-emission tomography and computed tomography scan revealed abnormal uptakes in numerous locations (e.g., the thyroid's isthmus and left lobe, bilateral parotid gland, subcutaneous tissues, etc.). The patient underwent palliative therapy, including total thyroidectomy, bilateral central neck dissection, left lateral neck dissection, and excision of the bilateral parotid and salivary gland. A whole-body scan post-therapeutic radioactive iodine ablation showed exclusive thyroid bed uptake. Subsequently, the patient underwent continuous thyroid stimulating hormone repression therapy and was treated with lenvatinib chemotherapy for ~8 months. The primary thyroid tumor, pancreatic metastasis, and cervical lymph node metastasis were both positive for *BRAF*^*V*600*E*^ and *TERT* promoter (C288T) mutations. After 13 months of follow-up, the patient is currently in stable clinical conditions. In conclusion, the present case is an extremely rare occurrence of simultaneous multiple RDMs from PTC as the initial presentation.

## Background

Thyroid cancer is the most common endocrine tumor, with an increasing incidence worldwide (1). Differentiated thyroid cancer (DTC), which includes papillary, follicular carcinoma and Hurthle cell carcinoma, comprises the vast majority of all thyroid cancers (2). Of note, papillary thyroid carcinoma (PTC) is the most frequent type of DTC (3). Compared with other thyroid malignancies, PTC is typically characterized by an indolent clinical behavior with good prognosis and long-term survival (3–5). While PTC commonly metastasizes to regional lymph nodes (6, 7), distant metastases may sparsely occur. However, the latter is extremely rare, especially multiple organs distant metastases (with the exception of the lungs and bones). Additionally, these metastases are predominantly identified in the course of follow-ups. Herein, we report the case of a patient who presented initially with synchronously multiple organs rare distant metastases (RDMs) from a diagnosed primary PTC.

## Case Presentation

**Figure 1:**
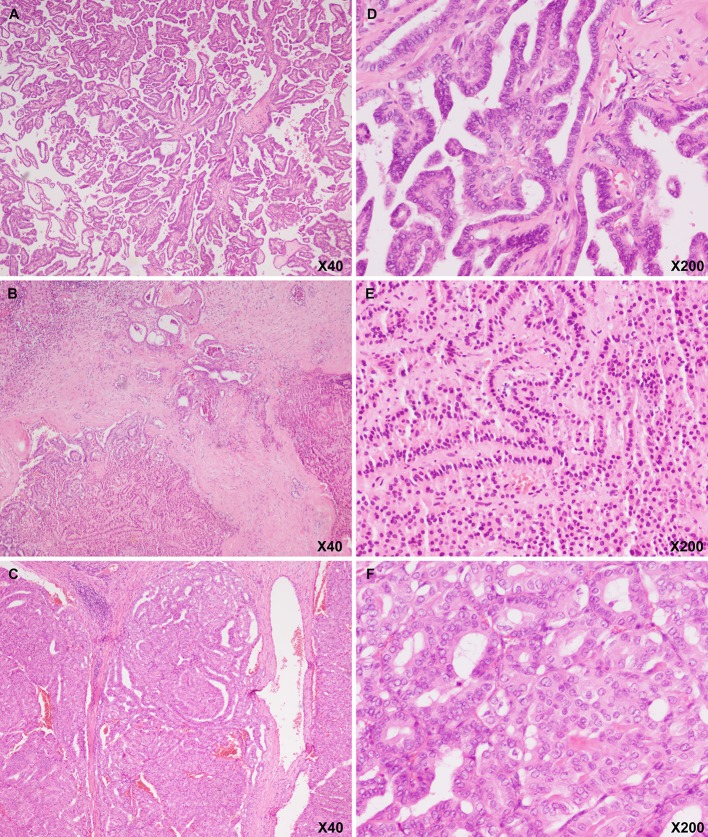
Hematoxylin and eosin image showing that the pancreatic body (**A**: magnification, ×20; **D**: magnification, ×200), liver (**B**: magnification, ×20; **E**: magnification, ×200), and diaphragmatic (**C**: magnification, ×20; **F**: magnification, ×200) masses had a papillary architecture with folded nuclei with grooves, which is a characteristic of thyroid papillary carcinoma.

A 48-year-old man with a recent history of epigastric pain was referred to the Department of Integrated Traditional Chinese & Western Medicine, West China Hospital, Sichuan University for the treatment of pancreatitis in January 2018. During the treatment, the patient underwent abdominal ultrasound examination that revealed a hypoechoic nodule (2.2 × 2.1 × 1.8 cm) in the pancreatic body, dilation of the main duct (the largest diameter: 0.6 cm) in the pancreatic body and tail, and slightly hyper-echoic intrahepatic nodule (diameter: 0.7 cm) in the right liver. Subsequently, the patient was transferred to the Pancreas Surgery Department of our institution and underwent resection of masses located in the pancreatic body, surface of the liver, and diaphragm in January 31, 2018. Intraoperatively, a firm irregular mass in the pancreatic body measuring 2.5 × 2.0 × 1.5 cm, three firm irregular masses on the surface of the liver measuring 0.5–0.8 cm, and an isolated firm irregular mass located in the diaphragm measuring 1.5 × 1.0 × 0.7 cm were observed. Unfortunately, the patient again underwent cholecystectomy, choleenterostomy, and enterolysis of prior surgery due to obstructive jaundice, which is considered a complication. Pathological examination of the masses' tissues revealed carcinoma cells aggregating in papillary architecture, with folded nuclei, cells were forming nuclear grooves (Figure 1). Immunohistochemical analyses showed tissue positivity for the following markers: TG, PAX-8, TTF-1, CK19, HBME-1, Galection-3, P53, WT-1, DPC4, CA19-9, and MUC1. On the contrary, the following markers were not expressed: MUC5AC, MUC6, and MUC2. Based on these findings, a diagnosis of metastatic PTCs was performed. However, the patient showed no positive symptoms and signs in the neck and head at that time. Subsequently, the patient underwent ultrasound examination and received an ultrasound-guided fine-needle aspiration (US-FNA) in the neck in February 13, 2018. Ultrasound examination showed a mass (8.1 ×5.1 ×4.1 cm) in the left lobe of the thyroid and several swollen lymph nodes in the left side of the neck, and US-FNA cytology revealed primary PTC.

**Figure 2:**
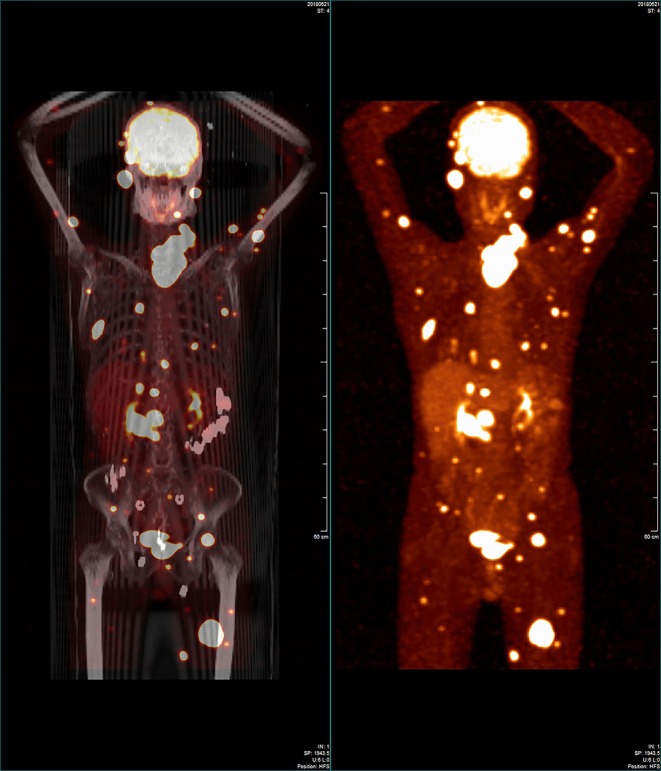
Whole-body 18-fluorodeoxyglucose positron-emission tomography/computed tomography image showing that some regions in the patient's body had widespread abnormal uptake.

An appointment was fixed with a thyroid surgeon from our institution in May 8, 2018. During the consultation, the patient presented with severe protein-energy malnutrition. After nutritional conditioning, in June 14, 2018, he was admitted to the Thyroid and Parathyroid Surgery Center for an evaluation prior to thyroid surgery. A physical examination performed in our department following postoperative recovery showed an irregular large nodule in the isthmus and left lobe of the thyroid, a swollen lymph node in the left cervical lateral compartment, and a mass in the right parotid gland. By nasopharyngeal laryngoscopy and gastroesophagoscopy, no abnormalities were observed. Ultrasound reexamination revealed the following findings: a very large hypoechoic mass (7.3 ×5.9 ×5.1 cm) in the isthmus and entire left lobe of the thyroid, multiple hypoechoic nodules (~0.2–0.3 cm) in the thyroid's right lobe, several swollen lymph nodes in the left cervical lateral compartment, several masses in the bilateral parotid region, several nodules in the bilateral salivary region, and a mass in the left gluteus maximus. US-FNA's histological results and immunohistochemical analyses confirmed the presence of metastatic PTCs bilaterally in the parotid gland, bilaterally in the salivary gland, and the left gluteus maximus. In order to further evaluate the situation and identify possible occult metastases, both an 18-fluorodeoxyglucose positron-emission tomography and computed tomography (18F-FDG-PET/CT) scan were ordered. The first revealed abnormal uptakes in the following locations: isthmus and left lobe of the thyroid, bilaterally in the parotid gland, left salivary gland, left cervical region, bilaterally in the lungs, pancreatic head, right kidney, and multiple cones, ribs, muscles and subcutaneous tissues (Figure 2). The patient's serum thyroglobulin (Tg) level was 303.00 ug/L (normal range: 1.4–78 ug/L). However, the serum free triiodothyronine, free thyroxine, Tg antibody, and thyroid peroxidase antibody levels were normal.

**Figure 3:**
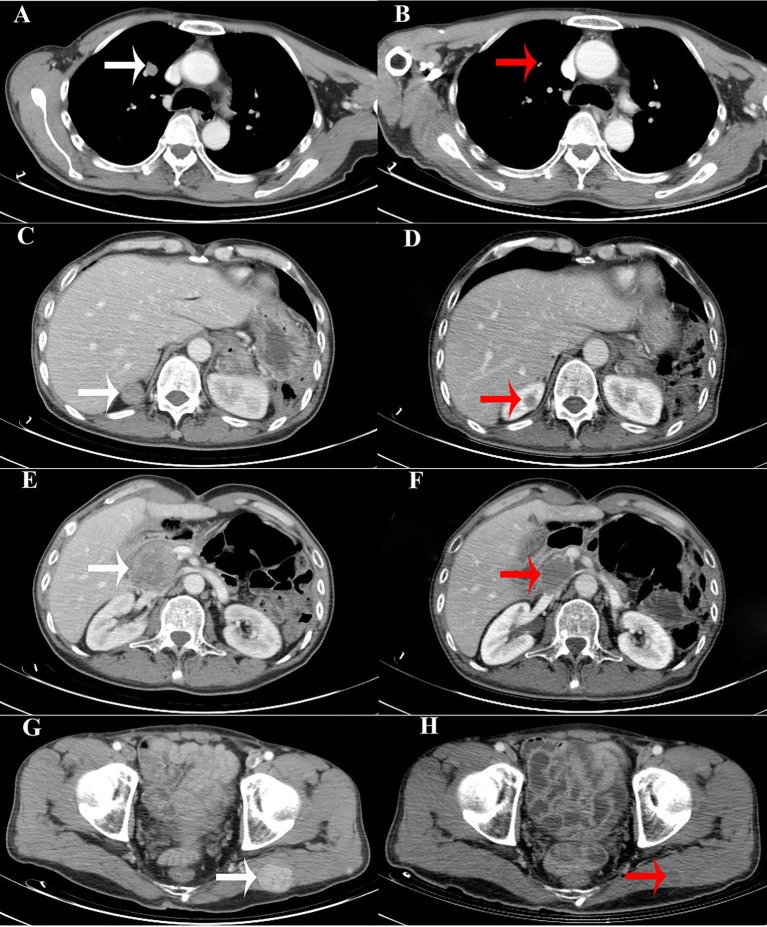
Computed tomography (CT) images showing that the size of the metastatic lesions had decreased. **(A,C,E,G)**. CT images of the metastatic lesions (white arrows) in the right pulmonary, right kidney, pancreatic head, and left gluteus maximus, respectively, before chemotherapy (lenvatinib). **(B,D,F,H)**. CT images of the metastatic lesions (red arrows) after chemotherapy (lenvatinib).

The patient underwent palliative therapy. Specifically, on July 2, 2018, the patient received a total thyroidectomy, bilateral central neck dissection, left lateral neck dissection, and bilateral excision of both parotid glands and salivary glands. Intraoperatively, a firm irregular tumor measuring 10.0 ×7.0 ×5.0 cm was observed. The tumor mass replaced the isthmus and left lobe of the thyroid. Additionally, it extended into the tracheoesophageal region and deepened into the superior mediastinum, invading the anterior cervical skeletal muscle. Furthermore, the tumor encapsulated and infiltrated the left recurrent laryngeal nerve and slightly infiltrated the left trachea's surface. Several swollen cervical lymph nodes were identified. The largest one measured 4.0 cm. Bilaterally in the parotid and salivary regions, there were many masses ranging from 0.5 to 3.5 cm. Of note, some of the masses invaded the facial nerve. Histological examination was performed post-operatively on the paraffin-embedded specimen. Results were consistent with PTC with multiple regional lymph nodes metastases and distant parotid and salivary metastases. The patient underwent thyroid stimulating hormone (TSH) repression therapy with sodium levothyroxine (Euthyrox), administered orally. In order to prepare for the radioactive iodine (RAI) ablation therapy, oral sodium levothyroxine treatment was suspended in August 2018. After 2 weeks, RAI ablation therapy with 200 mCi of I-131 was administered. A whole-body scan (WBS) following therapeutic RAI ablation showed uptake only in the thyroid bed and no uptake in any of the metastasizing lesions. Subsequently, the patient underwent TSH repression therapy with oral sodium levothyroxine. Additionally, he was treated with lenvatinib chemotherapy from December 2018. After ~8 months of treatment, numerous metastatic lesions had lessened on computed tomography (Figure 3). Additionally, serum thyroglobulin had decreased to 15.84 ug/L (from 313.60 ug/L) as of August 6, 2019. Currently, the patient is alive with no apparent symptom.

In order to complete PTC's assessment in this case, next-generation sequencing (NGS) was performed by lon PGM System with 1198X average sequencing depth. The goal was to identify gene rearrangements and genetic mutations detected in both the primary thyroid neoplasm and the metastasis in the cervical lymph node. Of note, results of our NGS demonstrated negativity for all of the following mutations: *ABL1, AKT1, ALK, APC, ATM, BRAF, CBL, CDH1, CDK4, CDKN2A, CHEK2, CSF1R, CTNNB1, DNMT3A, EGFR, ERBB2, ERBB3, ERBB4, EZH2, FBXW7, FGFR1, FGFR2, FLT3, GNA11, GNAS, HNF1A, HRAS, IDH2, JAK1, JAK2, JAK3, KDR, KIT, KRAS, MET, MLH1, MPL, NFE2L2, NOTCH1, NPM1, NRAS, PAX5, PDGFRA, PIK3CA, PPP2R1A, PTCH1, PTEN, RAF1, RB1, RET, SF3B1, SMAD4, SMARCB1, SMO, STAT3, STK11, TP53, U2AF1*, and *VHL*. Only *BRAF*^*V*600*E*^ mutation was positive. Furthermore, we identified a *TERT* promoter mutation (C288T) by means of additional testing in the primary thyroid tumor, the pancreatic and cervical lymph node metastasis.

## Discussion

For PTC, the frequent metastases are regional lymph node metastases. However, distant metastases are infrequent. Once distant metastases develop, PTC's prognosis becomes poor. Generally, PTC's distant metastases are located in the lungs and bones. Additional locations have only been sporadically reported (8–17), and are rare. In the present study, we describe the metastasis of distant organs/organization other than the lungs and bones as RDM. Here we report an extremely rare case of multiple simultaneous RDMs as PTC's initial presentations.

The patient only underwent abdominal ultrasound examination, and other imaging examinations were not performed prior to the first operation. The presence of any mass or nodule on the surface of the liver and diaphragm was not observed on abdominal ultrasound examination. These masses were incidentally found during operation probably because they were extremely small to be detected on ultrasound examination. All resected masses were confirmed as metastatic PTCs via pathological examination and immunohistochemical analyses. Subsequently, the surgeon focused on the patient's thyroid problem. Ultrasound examination showed a large mass in the left lobe of the thyroid, and US-FNA cytology revealed primary PTC. This finding indicated that the primary PTC may have been present for a long time. However, the patient did not present with positive signs and symptoms in the neck and head before ultrasound examination, which may be attributed to the fact that the mass originally extended into the tracheoesophageal region and deepened into the superior mediastinum. Therefore, early thyroid imaging may be necessary for this condition. As the patient developed the complication, he was admitted in the Pancreas Surgery Department for 39 days. In addition, it was difficult for the patient to fix an appointment with a thyroid surgeon in our institution due to more number of patients. Therefore, it took some time for the patient to be finally admitted at the Thyroid and Parathyroid Surgery Center for thyroid surgery. In our center, the second thyroid ultrasound examination and physical examination indicated that the mass was transversely larger and had extended to the isthmus of the thyroid. In addition, other multiple RDMs were found. Meanwhile, the primary PTC may have metastasized to other parts of the body. After referring to the detailed medical record of the patient, abdominal CT scan after the first operation showed several small masses in the upper segment of the right posterior lobe of the liver and a mass in the upper pole of the right kidney. Compared with the previous CT scan, the latest CT scan conducted ~5 months earlier showed that the size of the masses, which are located in the upper segment of the right posterior lobe of the liver and in the upper pole of the right kidney, increased. The two masses and the resected masses were considered as metastatic lesions upon initial presentation. It cannot be ruled out that the primary PTC metastasized to other organs and/or tissues upon initial presentation. However, no radiological images could support this assumption. The occurrence of multiple RDMs is normal at the late stage of poorly differentiated thyroid carcinoma. However, the pattern of poorly differentiated thyroid carcinoma was not found in the primary tumor, metastatic lymph nodes, and metastatic lesions in other distant locations. Therefore, PTC may have transformed into poorly differentiated thyroid carcinoma.

Earlier reports have shown RDM locations of PTC. These include the following: liver, pancreatic, spleen, kidney, adrenal gland, brain, cerebellar, skeletal muscle, parotid gland, skin, and so on (8–17). In the present case, the RDM locations included liver, pancreas, kidney, parotid gland, salivary gland, multiple skeletal muscles, and subcutaneous tissues. Generally, locations of simultaneous multiple RDMs do not exceed three (10, 11, 18–23). Interestingly, in the present case, pathologically confirmed RDMs locations from PTC were six. These did not include possible kidney and subcutaneous tissues metastases by 18F-FDG-PET/CT. Our case was rarer. Specifically, in this case, metastases affecting multiple skeletal muscles included the diaphragm. Such finding was confirmed by both surgery and pathological examination. In an earlier literature review by Herbowski L, it was shown that the metastasis in the skeletal muscles did not include the diaphragm (24). To the best of our knowledge, this study is the first to show the presence of distant diaphragmatic metastasis from PTC. Diaphragmatic metastasis may be attributed to tumor foci on the liver surface because they are extremely close to the diaphragm. Our intraoperative finding showed three masses on the surface of the liver located below the capsular of the liver. The masses were relatively small, and no adhesion to the diaphragm was observed. The mass in the diaphragm was larger than the masses in the liver. The masses on the surface of the liver and diaphragm were not observed in preoperative abdominal ultrasound examination. Therefore, diaphragmatic metastasis was not likely caused by tumor foci on the liver surface. Based on a retrospective review, the majority of RDMs occur solitarily and sequentially. On the contrary, multiple simultaneous RDMs were extremely rare. However, skeletal muscle metastases were generally identified in multiple locations (24). Additionally, skeletal muscle metastases are generally accompanied by multiple other rare distant metastases (10, 23). Such finding may be linked to the fact that skeletal muscles have numerous lumps and are located in most of the body. The RDMs mostly occurred and were identified during the course of follow-up, subsequently to a thyroid surgery. Multiple simultaneous RDMs as the initial presentation of PTC are a rare event. The majority of the RDMs were identified post-operatively, while they were scarcely ever identified pre-operatively. It has been shown that among 1200 thyroid carcinoma patients treated in Czepczynski R's institution, fewer than 1% had distant metastases detected at the time of the initial diagnosis (25). Of note, a literature review by Davidson M showed that almost all PTC's pancreatic metastases were post-operatively identified (26). Additionally, an extensive literature review by Herbowski L showed that only 3/31 patients were pre-operatively identified to have PTC's skeletal muscle metastases (24).

According to published meta-analyses of PTC, *TERT* promoter, and *BRAF*^*V*600*E*^ mutations were associated with aggressive clinicopathological features (27, 28). The following are examples of such features: extrathyroidal invasion, lymph node or distant metastasis, advanced TNM stage, disease persistence or recurrence, and disease-specific mortality. Additionally, studies performed on PTC have shown that coexistent *BRAF*^*V*600*E*^ and *TERT* promoter mutations have a synergistic effect on poor clinical outcomes (29). In the case presented here, *TERT* promoter and *BRAF*^*V*600*E*^ mutations were simultaneously detected in the primary tumor. In addition, the patient had some aggressive clinicopathological features (i.e., extrathyroidal invasion, lymph node metastasis, distant metastasis, advanced TNM stage, and disease persistence). PTCs are the most common endocrine cancers and are usually associated with good survival. However, some variants of PTC, such as hobnail, tall cell, columnar, and solid variants, may behave more aggressively than classic PTC (30). According to the gene mutations and aggressive clinical behavior of our case, the PTC's subtype may be an aggressive variant, and the patient may have a poor prognosis. Unfortunately, PTC's histopathological variants were not routinely diagnosed at our institution, leaving the PTC's subtype of the present case unidentified.

Surgery, radiotherapy, and RAI ablation therapy have been used for the treatment of PTC's distant metastases with variable results. To date, the best therapeutic option is resection, followed by RAI ablation therapy. For the present case, although distant metastases could not be completely resected, total thyroidectomy was performed for preparation for possible RAI ablation therapy. Unfortunately, because the WBS post-therapeutic RAI ablation was negative for the metastasizing lesions, the RAI ablation therapy was useless for the treatment of distant metastases. Moreover, the patient did not receive radiotherapy for the treatment of several metastatic lesions in several parts of the body (Figure 2). Therefore, treatment with lenvatinib, a tyrosine kinase inhibitor with antiangiogenic properties, was initiated for distant metastases. Lenvatinib has been recently approved by the Food and Drug Administration and European Medicines Agency. It is a drug used to treat radioiodine-refractory DTC (RR-DTC). Lenvatinib demonstrated a superior progression-free survival over placebo among patients with RR-DTC in a phase III clinical trial (SELECT) (31). Additionally, it represents the most cost-effective treatment compared with sorafenib and placebo (32). In the present case, following lenvatinib chemotherapy, the multiple metastatic lesions shrunk significantly and his Tg levels gradually decreased from 313.60 to 15.84 μg/L. The patient survived progression-free during the periods of follow-up. This result suggests that lenvatinib is effective for the treatment of radioiodine-refractory PTC with multiple RDMs. Sugino K has investigated the clinical factors correlated to the efficacy of lenvatinib and showed that the presence of tumor-related symptoms is an independent predictor of poor progression-free survival and overall survival in patients who are on lenvatinib treatment for RR-DTC (33). Because the patient did not present with tumor-related symptoms during treatment with lenvatinib and was able to continue with the treatment without withholding it for long intervals, he is likely to achieve long-term survival. In addition to lenvatinib, the use of sorafenib, another kinase inhibitor, was approved for DTC in 2013 based on favorable outcomes observed in a phase 3 clinical trial (DECISION) (34). Other novel drugs, including dabrafenib, a selective BRAF inhibitor (35), and vemurafenib (36), have been assessed, and results showed that these drugs are promising for the treatment of *BRAF*-mutated papillary thyroid cancer. The patient in the current study may also benefit from these drugs. However, further clinical trials should be conducted to validate the efficacy of such treatment.

## Concluding Remarks

We reported an extremely rare case of simultaneous multiple RDMs originating from a PTC. Importantly, the multiple RDMs represented the initial presentation. This condition required complex management. The case presented here provided us experience in managing this entity. However, additional future studies are needed to help us recognize and appropriately manage this condition.

## Data Availability Statement

The datasets generated for this study are available on request to the corresponding author.

## Ethics Statement

The study protocol was approved by the Ethics Committee of Sichuan University and written informed consent was obtained from participants.

## Author Contributions

JY, YM, and RG managed the case. JY, YM, and YG collected patient data and image. JY wrote the manuscript. RG, ZL, and JZ conducted the study and revised the manuscript. All the authors approved the final manuscript and agreed to be accountable for the content of the work.

### Conflict of Interest

The authors declare that the research was conducted in the absence of any commercial or financial relationships that could be construed as a potential conflict of interest.

## Abbreviations

PTC: Papillary thyroid carcinoma
RDMs: rare distant metastases
US-FNA: ultrasound-guided fine-needle aspiration
TSH: thyroid stimulating hormone
DTC: Differentiated thyroid cancer
18F-FDG-PET/CT: 18-fluorodeoxyglucose positron-emission tomography and computed tomography
Tg: thyroglobulin
RAI: radioactive iodine
WBS: whole-body scan
NGS: next-generation sequencing
RR-DTC: radioiodine-refractory DTC.
